# Blood-Making

**Published:** 1887-08-27

**Authors:** 


### The Doctor's Pulpit.

### BLOOD-MAKING.

Blood-making is a much more important business than blood-letting. The latter had a temporary reputation as a method of curing disease ; the former is a process as old as the animal creation, and as indispensable as food or air. Nobody knows what life is, but everybody understands that if all or even a good part of the blood be run out of a man's body his life runs out at the same time. The writers of ancient Egypt did not know anything about the circulation of the blood, or if they did, th y did not commit their knowledge to writing, and so their secret was lost. They did know, however, that in some way or other the blood was indispensable to life. Moses prohibited the Israelites from eating the blood of animals, on pain of death ; for, said he, that would be eating the "life." It is enough that the flesh be for the sustenance of man ; do not at the same time eat the life itself.

The blood is manufactured in the body and by the body, daily. The white cells, the red cells, and the fluid are constantly being renewed. If the blood ceases to be made in sufficient quantity and of good quality the physiological policeman will soon be on the track, and pull up the owner of the factory in some way or other. Modern physiologists, those rare pioneers who track out the secrets of the animal organism with a patience and persistence beyond all praise, have tried and tried again to get into the most secret places of the factory where the blood is actually made. No emissary of a foreign power, bent upon discovering the methods employed by his enemy in the manufacture of death-dealing weapons, ever strove with greater patience and subtlety to accomplish his object than have the German, the English, the French, and the American physiologists to find out with certainty the organs which make the blood and the *modus operandi* of its production. For the blood has a definite structure ; and we know that it is constructed in and by the machine which we call the animal body ; why therefore should we not be able to put our finger on the actual organ or organs which make it, and say : "It is made *here ;* and this is exactly *how* it is done ? Many patient and persistent workers have toiled manfully, and have deserved more success than has fallen to their lot. For beyond the discovery of the fact that the spleen and the marrow of certain bones appear to be directly concerned in the manufacture of blood, they do not seem to be able to proceed with any confidence. Is that not a pity ? It would be if the actual manufacture of the blood depended upon our knowing how it is made ; but it does not depend on anything of the kind. The blood continues to be manufactured, us d up, and renewed in entire disregard of the physiologist.

There are differences in the qualities of blood. Like other things it may be "good, bad, or indifferent." There is such a thing as "blue blood," too, and the man who has most of it is of the least value in the physiological market. The fat, puffing, panting, wheezing, blue-lipped, and purple-faced alderman is the most typical specimen of the blue-blooded man. Poor old fellow : we will speak very gently of him, for he may not have many days to live. Nobody should wish to have too much "blue blood." The crimson fluid is the best, and everybody should try his utmost to lay in an abundant stock of that, and should make a point of keeping his physiological warehouse always full of it.

But did we not say just now that the man of science had not been able to find out exactly the process by which the blood is made : and if the man of science cannot find that out, how can the man of no science do anything at all towards keeping his own physiological warehouse well supplied with red blood of the very primest brand ? It is one of the easiest things in the worl l for a healthy man, even though he knows nothing whatever about the method of blood-making, to make blood, and to make it in sufficient quantity, and of the very best kind. No preliminary apprenticeship is required for this trade. What are the principal ingredients of the best quality of blood ? Wholesome food, pure water, and pure air. What is the best method of manufacture? The ingredients must be put into the animal "mill," and if the machinery be moderately and evenly worked in all its parts for a sufficient length of time every day, the result in a healthy man or woman will be the manufacture, without trouble at all, of a sufficient daily quantity of the best and most wholesome blood : blood, which, like "wisdom," is "better than gold"; blood, which now, as in the days of Moses, is recognised as the very life itself.

The farm-labourer, who does so many other things of prime importance, is a blood-maker of the very first order. The fashionable woman, who is so useless in almost every other respect, is generally also very useless at this most vital business. Women, indeed, are not nearly such good blood-makers as men, and especially women who live in towns. They really ought to be better, and nature intended that they should be, for she makes greater demands upon their stores. It is easy to see how women are such poor blood-makers, when it is remembered that the principal ingredients in its manufacture are good food, pure air, and pure water. For women, though they may take good food, seldom take enough of it ; and as for pure air, they often take none at all. Of course the business of women is largely indoors ; but if they had a little more practical intelligence they would surely try to counterbalance the inevitable deficiency of fresh air thus caused by insisting upon a daily period of exercise and full respiration in the purest outdoor atmosphere they could find. A few women do this, but the majority, even of well-meaning and fairly intelligent mothers, sisters, and daughters, have not the energy and determination of character to maintain a steady and all-the-year-round habit of spending a long period of each day in a pure atmosphere.

But what is the use of putting good ingredients into any mill or machine unless the mill is made to work? There must be motive power, and enough of it. There is a certain amount of motive power in the body, we grant, but this power requires to be added to by vigorous and abundant exercise every day. If corn be taken to a windmill, the quantity of flour turned out in a given time will, *cæteris paribus*, be strictly proportioned to the volume and velocity of the wind blowing. Just so, if good food and pure air and water be supplied to the animal body, will the quantity and quality of the blood made in a given time depend upon the energy of the physiological processes, and these again upon the due exercise of the whole body. If there be little wind the mill-sails will creep slowly and idly round, and the flour when made will be small in quantity and ill-dressed and finished. Strictly similar will be the result of want of energetic exercise in the animal body: the physiological mills will grind slowly and grind badly, and the resulting blood will be small in quantity and of a second or third-rate quality.

Nature is kind. Notwithstanding the demerits of some people as blood-makers she does not compel them to live, or starve, on the stores they are able to make by the natural processes which have just been described. She has, hidden in the earth, a certain substance, which, if taken into the human body, seems to set all the blood-making organs in motion at more than express speed. That substance is iron. A considerable quantity of iron can be extracted from the blood of animals by chemical processes. The addition of iron to the blood of persons needing it, seems to act like a top-dressing to feeble corn in warm rainy weather. Given the suitable dressing and the warm rain, the dwindling corn will in two or three weeks spring up on all hands as if by magic. Exactly so will a suitable preparation of iron act on a bloodless person, other things being equal. The breathlessness will cease, the weakness pass away, the pallor of the face be replaced by a soft warm flush, and the miserable invalid become strong, active, and full of bustling happiness. There are numerous preparations of iron, and only a skilful medical man can decide what is best for particular cases. The acetate will suit one, the ammonio-citrate another. The perchloride is a general favourite, and well deserves the esteem in which it is held. For delicate women, perhaps the best preparation of all is what is known as "Blaud's pill." It is made as follows: two-and-a-half grains of the dried sulphate of iron: two-and-a half grains of carbonate of potash: mixed with mucilage tragacanth. This pill is difficult to make, and is besides very strong, and should only be taken under the doctor's supervision. But if two be taken after each meal for an adequate length of time, in eight cases out of ten most satisfactory results will be sure to follow. One word more: in taking iron the points to observe are, that a suitable preparation must be selected: that it must be taken in sufficient quantities: and that it must be continued for a sufficient length of time. He or she who, needing iron, will not persevere with its use, will certainly not reap the reward, and as certainly will not deserve to reap it. Half measures, here as elsewhere, bring discredit upon their authors and disappointment upon their users.

